# Refractive prescribing for preschool children by optometrists in England

**DOI:** 10.1111/opo.13050

**Published:** 2022-09-13

**Authors:** Amy L. Thompson, Miriam L. Conway, Irene Ctori, Rakhee Shah, Catherine M. Suttle

**Affiliations:** ^1^ Division of Optometry and Visual Science School of Health and Psychological Sciences City, University of London London UK

**Keywords:** children, England, optometry, prescribing, refractive error

## Abstract

**Purpose:**

Correction of refractive error in children is important for visual and educational development. The aim of this questionnaire‐based study was to explore paediatric refractive correction by optometrists in England.

**Methods:**

An online questionnaire was piloted and distributed to optometrists in England. The questionnaire asked about respondents' characteristics (such as type of practice), management of refractive error in 1‐ and 3‐year‐old children and sources of information used as a basis for decisions on prescribing refractive error in children.

**Results:**

Two hundred and ninety‐three questionnaires were returned, although only 139 (47%) were fully completed. In an average month, about half of respondents examined no children between 0 and 2 years of age, and about half examined no more than five children aged 3–4 years. A significant proportion indicated they would refer children aged 1 or 3 years with refractive error and no other signs or symptoms into the hospital eye service. Almost a quarter would prescribe in full or in part an isometropic refractive correction of +2.00 D for a 3‐year‐old (within the normal range) with no other signs or symptoms, suggesting a degree of unnecessary prescribing. Almost all would act in cases of clinically significant refractive error. Respondents made similar use of their colleagues, optometric or postgraduate/continuing education, professional guidance and peer‐reviewed research as sources of evidence on which to base decisions about prescribing for paediatric refractive errors. Most reported ‘never’ or ‘rarely’ using Cochrane reviews.

**Conclusions:**

These results suggest optometrists often defer management of paediatric refractive error to the hospital eye service, with implications in terms of underutilisation of community optometric expertise and burden on the National Health Service. In some cases, the results indicate a mismatch between respondents' reported management and existing guidance/guidelines on paediatric prescribing.

**Key points**

- In a typical month, about half of the optometrists in this cohort examined no children aged 0–2 years, and fewer than five children aged 3–4 years, in agreement with previous findings that community optometrists examine few young children.
- A significant proportion of optometrists in this cohort indicated that they would refer children (aged 1 or 3 years) with refractive error and no other signs or symptoms to the hospital eye service, suggesting that optometrists' core skills in refractive correction may be underutilised in these cases.
- Almost no respondents reported using the Cochrane database as a basis for decisions on refractive prescribing in children. However, information from Cochrane reviews may reach them via other sources such as continuing education.

## INTRODUCTION

The correction of refractive error in childhood is important for at least two reasons. First, normal, equal stimulation of each eye is needed for the visual system to mature normally and to avoid amblyopia,^1^, ^2^ a condition which may be treated in early childhood^3^ but is less amenable to later treatment.^4^ Second, uncorrected refractive error may have a negative impact on preschool literacy,^5^ whilst spectacle wear at school‐entry age has the potential to improve literacy.^6^

The optometrist or ophthalmologist considers the need for refractive correction for each child on an individual basis. This clinical decision may be based on experience and knowledge gained through optometric and postgraduate education^7^ and may be supported by clinical guidelines or other sources of advice or evidence. Guidelines may, in turn, be based on experience and pooled knowledge, devised by consensus and may or may not be based in part on research evidence.

A survey of hospital‐based optometrists in the United Kingdom indicates that most would prescribe a partial correction, reduced from the full refractive error.^8^ Similar surveys have been conducted amongst optometrists or ophthalmologists in the United States,^9^, ^10^ India,^11^ Saudi Arabia^12^ and amongst community and hospital optometrists in the United Kingdom.^13^ However, the reasons for this practice are unclear, and there are currently no data on the prescribing practices of community‐based optometrists in the United Kingdom. This study used a questionnaire to ask optometrists about their refractive prescribing habits in preschool children and the basis for their decision‐making.

## METHODS

An online questionnaire (see Appendix S1) was developed using Qualtrics (qualtrics.com) and was piloted by nine optometrists including five working in independent practices, three in multiple practices and one locum optometrist. These optometrists were contacted via the Cheshire Local Optical Committee (LOC), and responses were anonymous. Following their feedback, questions were organised into three sections as follows:

Section A: Information about the type and number of years of practice, postgraduate qualifications, the number of children seen in an average month who were 0–2 years and 3–4 years of age and the percentage of children in each age group on whom the respondent would routinely perform a cycloplegic refraction.
Section B: Information about the action the optometrist would take (to prescribe the full or reduced correction, refer or no action) for children aged 1 and 3 years with hyperopic, myopic or anisometropic spherical errors and astigmatic errors.
Section C: Information about the sources of information used by the respondent as a basis for spectacle prescribing in preschool children.

The web link for access to the questionnaire was made available to all LOCs in England via the LOC Support Unit. There are 78 LOCs in total, but the number of members within each is not public. The LOC Support Unit estimated that secretaries would have correct email addresses for about 100 optometrists in each LOC (~7800 email address in total). Note that we had no control over LOCs sending the link to their members, so we do not know the number of members who received it. However, if all members received the link, then an estimated response rate of 15% would provide 1170 responses.

The study was approved by the City, University of London Optometry proportionate review ethics committee.

Descriptive statistics were used to assess the proportions of respondents in each type of practice (e.g., independent), or with a postgraduate qualification, the average number of years in practice, the proportion testing children in practice at different levels of frequency, using cycloplegic refraction in children and using different sources of information to underpin paediatric refractive prescribing decisions. Chi‐squared tests were used to look for categorical associations between paediatric experience and both practice type and cycloplegic refraction, as well as between the number of years in practice and sources of information. A Spearman rank order test for correlation was used to explore the strength of the relationship between the number of years in practice and the likelihood of administering cycloplegic refraction. A Kruskal–Wallis Test was used to compare any differences between the frequency of cycloplegic refraction and either self‐declared paediatric experience or practice type. *Post*‐*hoc* analysis with Bonferroni correction was then carried out to determine any pairwise comparisons; *p*‐values of <0.05 were considered statistically significant.

## RESULTS

Two hundred and ninety‐three online questionnaires were returned. The total number of optometrists made aware of the questionnaire is not known, but the estimated maximum was 7800 (as explained earlier). If all the LOC members were aware, then the response rate would be very low, at 3.8%. Only 139 (47%; 1.8% of the estimated maximum number of potential respondents) of these were fully completed. All available responses were included in the analysis.

### Practice‐related data (section A)

The respondents' dominant practice type (in which they spent 60% or more of their working hours over the past year) was 27% multiple practice, 42% independent practice, 4.4% locum work and 4.1% hospital practice. Note that 22.5% of responses to this question were not included in the analysis due to response error. Specifically, the question asked respondents to indicate the percentage of their working hours spent in each type of practice, and to ensure that these summed to 100. In 22.5% of cases, the percentage allocation did not sum to 100. Respondents had been in practice (excluding significant periods of leave) for a mean of 21 years (range 1–55; SD 12 years).

Of 244 respondents who answered the question ‘do you have a postgraduate qualification or previous experience specific to paediatric optometry’, 209 (86%) answered ‘No’ and 35 (14%) ‘Yes’. Of the latter, 18 had experience in a hospital eye department, 10 had a MSc degree, diploma or certificate including paediatric optometry, three had research experience related to paediatric optometry (two at PhD level), three were behavioural optometrists or had experience of managing patients with visual stress and one was not specified. A significant association was found between dominant practice type and self‐declared paediatric experience (*X* ^2^ [1, *n* = 244] = 18.6, *p* = 0.002), respondents predominantly working in hospital practice being more likely to declare paediatric experience than those in independent, multiple or locum practice. This is not a surprising finding, since a high proportion of community optometric practices in England do not offer eye examinations to young children,^14^ whilst paediatric eye care is an integral part of hospital practice.

**FIGURE 1:**
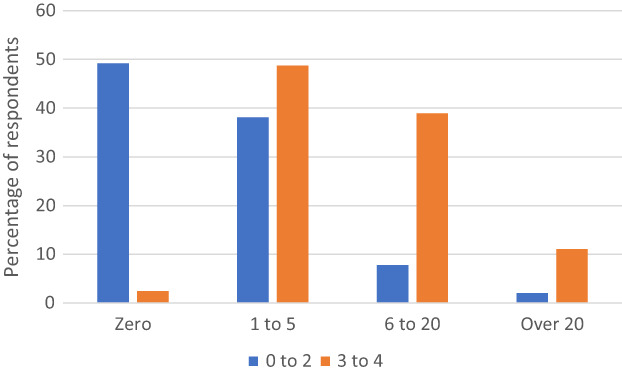
Percentage of respondents who indicated that they saw zero, 1 to 5, 6 to 20 or over 20 children aged 0–2 years or 3–4 years in an average month.

The questionnaire asked for a category‐based estimate of the number of 0–2 and 3–4‐year‐old children the respondent would examine in an average month. About half (49.2%) of the respondents to this question indicated that they examined zero children aged 0–2 years per month (Figure 1). A similar proportion (48.8%) indicated that they examined 1–5 children aged 3–4 years per month. Almost none of the respondents examined zero children aged 3–4 years in an average month.

Respondents who reported examining children in these age groups indicated that cycloplegic refraction is routinely performed on average (mean) in 44% of 0–2 year‐olds and 46% of 3–4 year‐olds.

Respondents with self‐declared paediatric experience were significantly more likely to carry out cycloplegic refractions in 0–2 year‐olds (Kruskal–Wallis *X* ^2^ [1, *n* = 152] = 5.03, *p* = 0.03). No significant association with paediatric experience was found for cycloplegic refractions in 3–4 year‐olds.

A significant negative correlation was found between the number of years in practice and the likelihood of administering cycloplegic refraction in the 0–2 and 3–4 year age groups (*r* = −0.26 and −0.31, respectively; *p* < 0.003). However, low *r* ^2^ values (7% and 10%, respectively) suggest that the number of years in practice is not a major explanatory factor for the use of cycloplegic refraction in these children.

Table 1 shows the median percentages of cycloplegic refractions in each age group and practice type. Respondents predominantly working in the hospital eye service (HES) reported the highest median percentage, followed by those in multiple, independent and locum practice. A Kruskal–Wallis test revealed a significant difference between the reported percentage of cycloplegic refractions carried out for 3–4 year‐olds in the four different practice types (*X* ^2^ [4, *n* = 211] = 25.29, *p* = 0.001). Bonferroni corrected *post*‐*hoc* analysis revealed significant differences between locum and multiple practice types (10.27, *p* = 0.01); locum and HES (19.30, *p* < 0.001) and independent and HES (12.89, *p* = 0.003). Despite a similar pattern of reported percentages in the 0–2 year‐old age range, no significant difference was found between practice types (*p* > 0.05), perhaps due to lower respondent numbers.

**TABLE 1:** Median percentage of cycloplegic refractions in 3–4 year‐olds reported by respondents predominantly in independent, multiple, locum or hospital practice

Dominant practice type	Median percentage cycloplegic refractions (*N* = number of respondents)	
	0–2 years (*N*, %)	3–4 years (*N*, %)
Independent	20 (80, 58.4)	30 (100, 51.0)
Multiple	50 (42, 30.7)	50 (73, 37.2)
Locum	23 (7, 5.1)	24.5 (12, 6.1)
Hospital Eye Service (HES)	99.5 (8, 5.8)	90 (11, 5.6)

### Prescribing patterns (section B)

#### Isometropic refractive error

**FIGURE 2:**
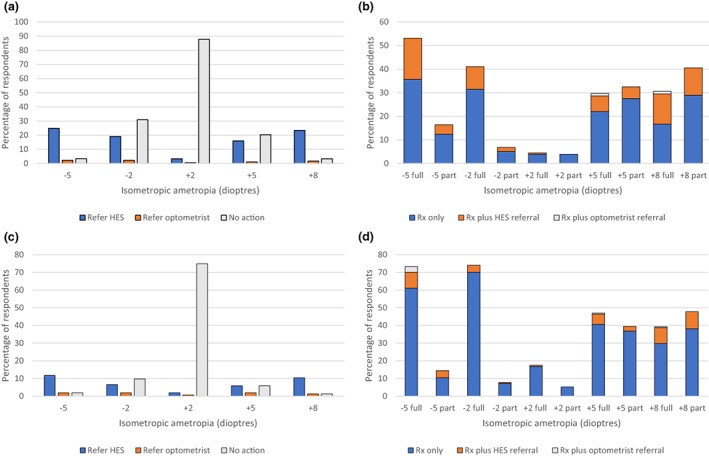
Percentage of respondents indicating the action they would take in the case of a 1‐year‐old (a, b) and 3‐year‐old (c, d) isometropic ametropic child with no significant history or signs, at their first eye examination. HES, hospital eye service. ‘Optometrist’ indicates another community optometrist. ‘Full’ and ‘part’ indicate prescription of the full or partial refractive correction.

**FIGURE 3:**
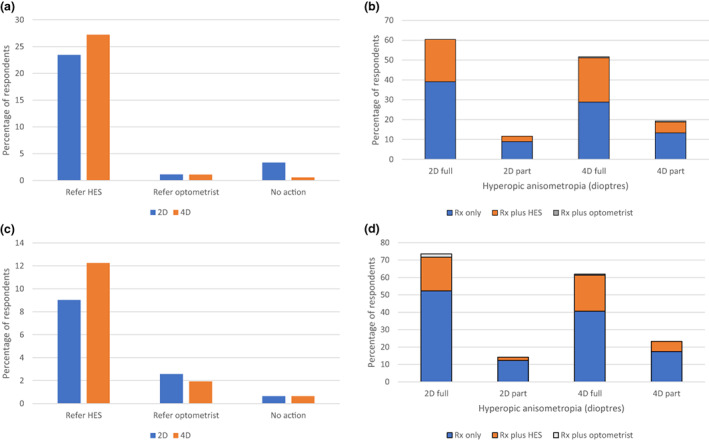
Percentage of respondents indicating the action they would take in the case of a 1‐year‐old (a, b) and 3‐year‐old (c, d) child with anisometropic hyperopia and no significant history or signs. HES, hospital eye service. ‘Optometrist’ indicates another community optometrist. ‘Full’ and ‘part’ indicate prescription of the full or partial refractive correction.

**FIGURE 4:**
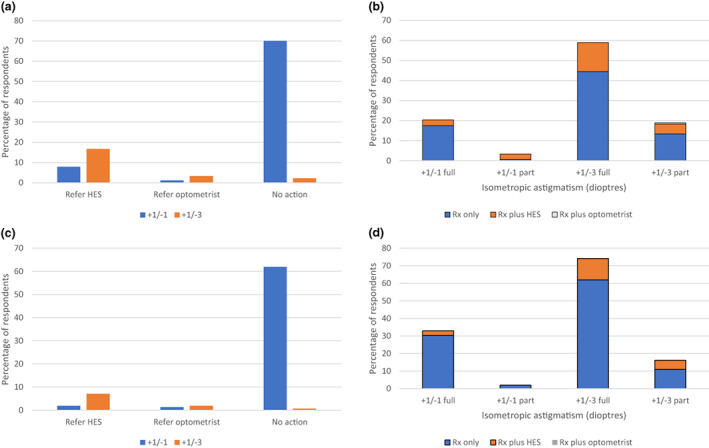
Action respondents would take in the case of a 1‐year‐old (a, b) or 3‐year‐old (c, d) with isometropic astigmatism of +1.00/−1.00 × 180 or +1.00/−3.00 × 180 and no significant history or signs. HES, hospital eye service. ‘Optometrist’ indicates another community optometrist.

The percentage of respondents who would prescribe a full or reduced refractive correction for a range of refractive errors in preschool children without significant history or signs is shown in Figures 2, 3, 4. The figures also show the percentage of respondents who would refer to the HES or to a community optometrist without prescribing or would take no action.

Note that in response to questions about each refractive error, between zero and 22% of respondents indicated that they would both prescribe (in full or part) and refer to the HES or a community optometrist. The proportion of respondents that would prescribe in full or in part and/or refer and that would take no action are stated in the text and depicted in the figures.

Figure 2a shows that most of the respondents (87.8%) indicated that they would prescribe no refractive correction and would not refer a 1‐year‐old isometropic 2D hyperope. The full correction (Figure 2b) would be prescribed by 4.4% (including 0.6% also referring to the HES and no referrals to a community optometrist) and a reduced correction would be prescribed by 3.9% (no referrals elsewhere). At the higher isometropic refractive error of +5.00 DS, 29.7% of respondents would prescribe in full (including 6.6% also referring to the HES and 1.1% to an optometrist) and a reduced prescription would be issued by 33.0% (including 5.0% also referring to the HES and 0.6% to an optometrist). At +8.00 DS, 30.6% would prescribe in full (including 12.8% also referring to the HES and 1.1% to an optometrist) and a reduced prescription would be given by 41.1% (including 11.7% also referring to the HES and 0.6% to an optometrist).

In the unusual scenario of a 1‐year‐old myope, at −2D 41.0% would prescribe in full (including 9.6% also referring to the HES and none to an optometrist) and reduced correction would be issued by 6.7% (including 1.7% also referring to the HES and no referrals to a community optometrist). At −5D, 53.2% would prescribe in full (including 17.5% also referring to the HES and no referrals to a community optometrist), whilst 16.4% would prescribe a reduced correction (including 4.0% also referring to the HES and no referrals to a community optometrist). The proportion who would refer to a community optometrist without prescribing was less than 2.5% for each of these refractive errors.

Similarly, in the case of a 3‐year‐old child, the majority of respondents (74.8%) would take no action in the case of 2D of isometropic hyperopia (Figure 2c). The full prescription would be issued by 17.4% (including 0.7% also referring to the HES and no referrals to a community optometrist), whilst 5.2% would prescribe in part (with no referrals elsewhere). At 5D, 47.1% would prescribe in full (including 5.8% also referring to the HES and 0.7% to a community optometrist), whilst 39.4% would prescribe in part (including 2.6% also referring to the HES and no referrals to an optometrist). At 8D, 39.4% would prescribe in full (including 9.0% also referring to the HES and 0.7% to an optometrist), and 47.7% would prescribe in part (including 9.7% also referring to the HES and no referrals to a community optometrist).

For a 2D isometropic 3‐year‐old myope, 74.0% would prescribe in full including 3.9% who would also refer to the HES and none to a community optometrist. 7.8% of respondents would prescribe in part and this figure includes 0.7% who would also refer to the HES and none to an optometrist. Referral to the HES without prescribing was indicated by 6.5%, whilst 1.2% would refer to a community optometrist without prescribing and 9.8% would take no action. For a 5D myope at this age, 70.8% would prescribe in full including 9.1% who would also refer to the HES and 0.6% who would refer to a community optometrist. 13.6% would prescribe in part, including 3.2% who would refer to the HES and no referrals to a community optometrist. Without prescribing, 11.7% would refer to the HES, whilst 1.9% would refer to a community optometrist and 1.9% would take no action.

For a 1‐year‐old child presenting with hyperopic anisometropia of 2D or 4D, very few of the respondents (3.4% at 2D and 0.6% at 4D) would take no action (Figure 3a). At each level of anisometropia, about a quarter of respondents (23.5% and 27.2%, respectively) would refer to the HES without prescribing, and about 1% would refer to a community optometrist without prescribing. At 2D, 60.3% would prescribe in full (including 21.2% also referring to the HES and no referrals to a community optometrist; see Figure 3b), and 11.7% would prescribe in part (including 2.8% also referring to the HES and no referrals to a community optometrist). At 4D, 51.7% would prescribe in full (including 22.2% also referring to the HES and 0.6% to a community optometrist), and 19.4% would prescribe in part (including 5.6% also referring to the HES and 0.6% to a community optometrist).

A similar pattern emerged for a 3‐year‐old child with the same levels of anisometropia, but in this case, a lower proportion would refer without prescribing (Figure 3c). At 2D of anisometropia, 73.5% would prescribe the full correction (including 19.4% also referring to the HES and 1.9% to a community optometrist; see Figure 3d), whilst 14.2% would prescribe in part (including 1.9% also referring to the HES and no referrals to a community optometrist). At 4D, 61.9% would prescribe in full (including 20.7% also referring to the HES and 0.6% to a community optometrist), whilst 23.2% would prescribe in part (including 5.8% also referring to the HES and no referrals to a community optometrist).

In the case of a 1‐year‐old with isometropic astigmatism of +1.00/−1.00 × 180, 70% of respondents would not prescribe or refer, whilst 8% would refer to the HES (Figure 4a). As shown by Figure 4b, 20.3% of respondents would prescribe in full (including 2.8% also referring to the HES and no referrals to a community optometrist) and 0.6% would prescribe in part (including no referrals elsewhere). At +1.00/−3.00 × 180, 58.9% would prescribe in full (including 14.4% also referring to the HES and no referrals to a community optometrist) and 18.9% would prescribe in part (including 5% also referring to the HES and 0.6% to a community optometrist).

For a 3‐year‐old with a refractive error of +1.00/−1.00 × 180, 32.9% would prescribe the full correction (including 2.6% also referring to the HES and no referrals to a community optometrist) and 1.9% would prescribe a partial correction (no referrals elsewhere). At the higher astigmatism of +1.00/−3.00 × 180, a small proportion of respondents would take no action or refer to a community optometrist without prescribing (0.7% and 1.9% respectively), whilst 7.1% would refer to the HES without prescribing (Figure 4c). A total of 74.2% would prescribe in full (including 12.3% also referring to the HES and no referrals to a community optometrist), whilst 16.1% would prescribe in part (including 5.2% also referring to the HES and no referrals to a community optometrist; Figure 4d).

A Chi‐squared test was conducted to look for relationships between prescribing patterns and self‐declared paediatric experience. After removal of all missing data (from respondents who did not answer questions on prescribing patterns; *n* = 142), small sample sizes in all categories initially prevented any statistical analysis. Data were therefore reduced into two categories based upon whether the respondent indicated that they would manage the patient themselves (including prescribe in full, a partial correction or no action) or refer elsewhere. Limited sample size still prevented analysis in most cases. However, for a 1‐year‐old child with isometric refractive error of +8.00 D, respondents with self‐declared paediatric experience were more likely to manage the patient themselves than those without this experience (*X* ^2^ [1, *n* = 151] = 3.9, *p* < 0.05, phi = 0.18). Similarly, for a 1‐year‐old child with 2D hyperopic anisometropia, clinicians declaring paediatric experience were more likely to manage the patient themselves than those without such experience. (*X* ^2^ [1, *n* = 151] = 5.1, *p* = 0.02, phi = 0.20).

### Reasons for partial correction

Respondents who indicated that they would not issue the full prescription were asked to explain why not and to state the reduced prescription. For each refractive error scenario, Table 2 shows the number of responses and the median reduction in the prescription. The results show that those not issuing a full correction of +5D to +8D to a 1‐ or 3‐year‐old child would reduce this by 1.50 D to 2.00 D on average, and a −5.00 D correction by 1.00 D to 1.50 D. Relatively few respondents indicated that they would reduce the 3.00 D cylinder (DC) component of an astigmatic prescription, by 1.00 DC on average. Similarly, few respondents indicated that they would undercorrect anisometropia of 2.00 D or 4.00 D by 0.75 D to 1.25 D on average. Note that, these are reductions in the magnitude of correction for the anisometropia and not a symmetrical reduction in the prescription which maintains the full anisometropic correction. The latter was indicated by some respondents who commented that the anisometropia needs to be corrected but the child may need a symmetrical reduction in the correction for each eye (for example, R + 1.00 DS L + 5.00 DS reduced to R plano L + 4.00 DS) to aid adaptation.

**TABLE 2:** The number (*N*) of respondents who indicated that they would issue a reduced refractive correction in each of the refractive scenarios and the median reduction in the prescription (D, dioptre) in each case

Refractive error category (D)	1‐year‐olds		3‐year‐olds	
	*N*/Total (%)	Median (range) reduction (D)	*N*/Total (%)	Median (range) reduction (D)
Isometropia				
+2	2/7 (28.5)	0.88 (0.75–1.00)	4/8 (50.0)	0.88 (0.50–1.00)
+5	31/60 (51.7)	2.00 (0.50–3.00)	39/61 (63.9)	1.50 (0.50–3.00)
+8	47/74 (63.5)	2.00 (0.75–5.00)	46/74 (62.2)	2.00 (1.00–4.50)
−2	4/12 (33.3)	1.00 (0.50–1.00)	6/12 (50.0)	0.75 (0.50–1.00)
−5	19/29 (65.5)	1.50 (0.50–4.00)	11/21 (52.4)	1.00 (0.75–3.50)
Anisometropia				
2	4 /21 (19.0)	1.00 (0)	2/22 (9.1%)	0.75 0.50–1.00)
4	10/35 (28.6)	1.25 (1.00–3.00)	7/36 (19.4)	1.00 (0.50–1.00)
Astigmatism				
1	0	N/A	0	N/A
3	15/34 (44.1)	1 (1.00–2.00)	10/25 (0.40)	1 (0.50–2.00)

*Note*: The numbers are stated as a proportion of the total number of respondents indicating that they would reduce the correction and as a percentage of this number. Note that, responses in the anisometropia category reflect reductions in the anisometropia itself and not symmetrical reduction in the right and left prescription.

Respondents who explained why they would reduce the prescription indicated that this was important for adaptation or to allow emmetropisation, with some respondents commenting instead that this question is difficult to answer without more information. For example, the question addressed each refractive error type (isometropia, anisometropia or astigmatism) without specifying the level, and did not specify the child's visual acuity.

### Sources of reference material (section C)

**FIGURE 5:**
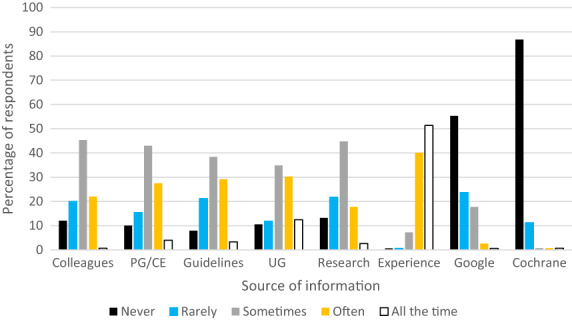
The percentage of respondents who never, rarely, sometimes, often or all the time use a range of sources of information or advice as a basis for decisions about spectacle prescribing for preschool children. Guidelines, College of Optometrists guidance; PG/CE, postgraduate or continuing education; UG = undergraduate (optometric) education.

One hundred and fifty‐two respondents answered the final question relating to the sources of information or advice on decisions about spectacle prescribing for preschool children (see Figure 5). Just under half (43%–45%) of respondents indicated that they sometimes consult colleagues, postgraduate or continuing education or peer‐reviewed research as a basis for their decision‐making. However, approximately one‐third (29%–34%) rarely or never use colleagues, College of Optometrists' guidance or peer‐reviewed research as a basis for their decision‐making. Forty‐two per cent of respondents use their knowledge from their optometric education often or all of the time, whilst a further 35% use this knowledge sometimes when making decisions on prescribing for preschool children. Almost none of the respondents make use of Cochrane reviews, with 98% indicating that they never or rarely refer to the Cochrane library for these decisions. It is worth noting, however, that the respondents may have accessed information derived from Cochrane reviews through other sources, such as continuing education. On the contrary, unsurprisingly, almost all (91%) respondents use their experience often or all of the time. More than three‐quarters (79%) of respondents never or rarely use an Internet search (using platforms such as Google) for decisions in this clinical area. No significant association was found between these sources of information and the number of years in practice.

## DISCUSSION

Appropriate correction of refractive error in early childhood is vital to avoid abnormal visual development and potential negative impact on academic development. Uncorrected hyperopic refractive error in a child could lead to manifest convergent strabismus, amblyopia and the permanent loss of binocular functions.^15^ Cycloplegic refraction is therefore an important part of the paediatric optometrist's armoury, allowing the full extent of the hyperopia to be unmasked, and is considered the gold standard for refractive error assessment in children.^16^ Undercorrected myopia is unlikely to cause amblyopia since the child is able to focus at near, but will cause poor vision at distance with implications for performance on a range of distance‐related tasks.

The results of this study show that optometrists in England with self‐declared paediatric experience or predominantly working in hospitals were more likely to administer cycloplegic refraction. These findings are consistent with previous survey data,^13^ and with a higher tendency amongst optometrists in hospital to use cycloplegia at children's first and subsequent eye examinations.^13^

Our finding that most respondents would take no prescribing or referral action in the case of 2D isometropic hyperopia in a 1‐ or 3‐year‐old is consistent with the fact that low‐to‐moderate hyperopia is normal in preschool children.^17^ Evidence‐based guidelines^18^ indicate that prescribing should be avoided for the child at both ages in this scenario. However, almost a quarter (23%) of respondents would prescribe this correction either in full or in part for the 3‐year‐old child, whilst 16% would refer the child into the HES without prescribing. Since the child in this scenario had no symptoms or signs and the refractive error is within normal limits, either course of action seems likely to incur unnecessary costs for the patient's parent or carer and for the National Health Service in terms of time, money and other resources.

The higher isometropic hyperopic refractive errors of 5 and 8D are both outside of the normal range at either 1 or 3 years. However, without signs or symptoms, it is not immediately apparent that a refractive correction needs to be prescribed. Guidelines by Leat^18^ indicate that the refractive error should be partially corrected, leaving a stimulus for accommodation. Results of a randomised controlled trial published subsequently, although inconclusive, suggested small to moderate or no benefit in immediately prescribing a correction for moderate hyperopia (+3 to +6D) in nonstrabismic 1‐ to 2‐year‐old children, compared with a delayed prescribing approach.^19^ A systematic review, also published after the guidelines, also concluded that the benefit of refractive correction for hyperopia (over +2D) in children aged up to 3 years of age is unclear.^20^ In this study, for children aged both 1 and 3 years, at least one‐third of respondents were in line with the 2011 guidance, the remainder prescribing in full, referring the patient or taking no action.

Referral is an interesting course of management, since it seems to imply that the referring optometrist deems the hospital better able to care for the patient by verifying the refractive error and prescribing any correction that may be required. The optometrist's core role has been refractive error measurement and correction for some time, but previous research indicates that not all optometrists offer eye examinations to young children,^7^, ^12^, ^13^ and instead refer them to colleagues or to hospital services. Our finding that a notable proportion of respondents would refer these patients without treatment suggests that they may not feel confident of their refractive findings, in prescribing to children in these scenarios or following them up, may feel that they do not have access to the appropriate equipment for eye examinations on young children, or a combination of these and other concerns. Referral to the HES fails to use optometrists' core skills in refraction and this underutilisation adds to the workload within the HES. Myopia development and interventions to slow its progression in childhood have attracted a great deal of research interest. A Cochrane systematic review of randomised controlled trials^21^ found moderate certainty evidence for the effectiveness of topical atropine with or without multifocal spectacles, or multifocal spectacles alone, as interventions to slow myopia progression in children. The guidelines by Leat^18^ indicate that at −2D and at −5D, myopia should be under‐corrected by about 0.5 to 1D and by 2D, respectively, to allow emmetropisation to occur. However, Walline et al.^21^ found low certainty evidence of an *increase* in myopia progression when undercorrected. Faced with these findings, the optometrist must choose between following guidelines with a risk of accelerated myopia progression or being guided by more recent high‐level evidence with a risk of disrupting emmetropisation. College of Optometrists guidance on this subject discusses the range of interventions and relevant evidence, the lack of knowledge about long‐term risks and indicates that the practitioner should offer the patient (or parent) an informed choice.^22^

Most of our respondents indicated that they would prescribe an isometropic myopic refractive correction either in full or in part for a 1‐ or 3‐year‐old, with or without additional referral. However, our questionnaire did not ask about the form of intervention. For example, the respondents may have considered multifocal contact lenses, but our results cannot reflect this.

In the case of significant anisometropia, most of our respondents would prescribe either in full or in part for a 1‐ or 3‐year‐old child. For the younger child, approximately one‐quarter would refer the child without prescribing. As discussed earlier, this raises questions about whether the respondents felt inadequately equipped or prepared to assess and prescribe a refractive correction for this young child.

Most respondents indicated that they would take no action in the case of a 1‐ or 3‐year‐old with isometropic astigmatism (+1.00/−1.00 × 180; mean spherical equivalent +0.5 D), consistent with guidelines stating that 1D of oblique astigmatism should be partially or fully corrected at this age, but not referring to horizontal or vertical astigmatism.^18^ Conversely, at a higher level of astigmatism (+1.00/−3.00 × 180), most indicated that they would prescribe the full refractive correction at both ages, with an additional smaller proportion prescribing in part, and few referring the patient without prescribing. These actions are not in complete agreement with the guidance by Leat^18^ that a reduced correction should be given at each age to allow emmetropisation to occur, whilst correcting the astigmatism during a critical period at the younger age, and based on empirical findings at the older age.

Responses from those who explained why they would reduce a prescription indicated that adaptation or emmetropisation were important factors. Undercorrection for these reasons is evidence‐based,^18^ but the level of undercorrection in this study ranged widely, for example, between reductions of 0.75 D and 5.00 D in a 1‐year‐old child with +8D isometropic hyperopia, and between 0.50 D and 3.00 D in a 3‐year‐old child with +5D isometropic hyperopia. This suggests a need for clearer recommendations on the level of reduction most likely to benefit young children.

Responses that are not consistent with high‐level evidence or with the most recent evidence‐based guidelines on prescribing in children raise questions about whether optometrists make use of such information to inform their clinical decision‐making. Responses were similar for the sources ‘colleagues’, ‘postgraduate or continuing education’ and ‘College of Optometrists’ guidance’, with about one‐quarter referring to these ‘often’ or ‘all of the time’ and fewer than one‐third using them ‘rarely’ or ‘never’. The College of Optometrists,^23^ referring to Leat,^18^ provides guidance on prescribing refractive correction for younger children, with advice to consider factors such as whether the refractive error is within the normal range, whether correction could affect emmetropisation and whether it will improve the child's vision.

About one‐third of respondents used their optometric education ‘often’ or ‘all of the time’, with fewer than one‐quarter of respondents using this source ‘rarely’ or ‘never’, consistent with previous findings that optometrists consider information from optometric and postgraduate education to be important sources for their clinical decision‐making.^7^

Unsurprisingly, almost all respondents indicated that they use experience ‘often’ or ‘all of the time’, whilst almost 80% of respondents ‘never’ or ‘rarely’ use a general search of the Internet (such as Google) to find information or advice as a basis for their decision‐making about prescribing in preschool children. Interestingly, 87% ‘never’ use the Cochrane library, whilst 11% use it ‘rarely’. Cochrane is a free, online library of high‐level evidence including high‐quality systematic reviews, trials and ‘clinical answers’ which are updated periodically to reflect current best evidence.^24^ The latter are intended to provide clinicians with rapid, easy access to evidence‐based answers to clinical questions, and Cochrane reviews include summaries making them both relatively digestible and easy to use at the point of clinical decision‐making. Cochrane is organised into groups based on clinical area, including an Eyes and Vision group which produces systematic reviews relating to a range of topics such as prescribing for myopic, hyperopic, strabismic and/or amblyopic children.^25^ Our finding that respondents very rarely use these resources may reflect a preference to use other sources of information or evidence, a lack of Cochrane evidence relating to their clinical questions on paediatric prescribing, or perhaps a lack of awareness of the Cochrane library. The findings suggest that efforts to increase optometrists' understanding of the benefits of Cochrane resources, and how to use them, may have advantages for both clinicians and patients. In the longer term, better quality primary research and an increase in the range of questions addressed by Cochrane reviews and clinical answers will also help to ensure that common clinical questions are addressed.

The UK optometric profession recognises the importance of eye care for children of all ages, as reflected by the College of Optometrists' information for patients.^26^ Our results showed that about half of respondents examined zero children aged 0–2 years, consistent with previous findings that many young children are not seen by optometrists.^14^, ^27^ Multiple factors may influence children's access to optometric care including the finding that almost one‐quarter of parents in Britain may not take their 4–16‐year‐old children for a sight test and may assume (incorrectly) that vision screening is provided for all children in schools.^28^ In addition, previous research shows that even if parents do attempt to book an appointment, only 22% and 50% of optometry practices in England offer eye examinations for children aged one or three, respectively.^14^ The scenarios in this study involving a 3‐year‐old child are more likely to relate to community optometrists' experience in practice, but the scenarios with both 1‐ and 3‐year‐old children reveal the extent to which optometrists in the United Kingdom are willing to manage young patients with refractive error and no other visual anomaly.

This survey was distributed to LOCs in the United Kingdom via the LOC Support Unit. However, we cannot be certain that all LOC members were made aware of the survey or had access to the questionnaire, so we do not know the questionnaire response rate. More importantly, the fact that it targeted only a subset of practicing optometrists in England (LOC members) and that it may not have been made available to all of that subset means that our data may be affected by selection bias. In addition, the study relies on self‐report rather than a review of records documenting paediatric refractive prescribing decisions. This is a limitation since the reported behaviour does not necessarily reflect the optometrists' true prescribing patterns. Another limitation, as pointed out by some of our respondents, is that the questions did not give sufficient information in some cases for the situation to be fully understood. This could be addressed in future research by including fewer, simpler scenarios with more complete information, or by use of an interview approach, allowing respondents to understand the scenarios fully and to explain their prescribing rationale. In addition, data on practice type from 22.5% of respondents were excluded due to error in using the sliding scale in the online questionnaire. This issue was not raised during the pilot phase, but clearly was problematic for some of the participants and led to loss of significant data on practice type.

The 293 responses we did receive represent about 2% of the approximately 14,100 optometrists practicing in England^29^ and therefore are unlikely to represent this population accurately. However, our distribution of dominant practice types is broadly similar to that of the College of Optometrists' Optical Workforce Survey, suggesting that our data reflect the types of practice in which optometrists work.^30^

## AUTHOR CONTRIBUTIONS

**Amy L. Thompson:** Conceptualization (equal); data curation (lead); formal analysis (equal); investigation (lead); methodology (equal); writing – review and editing (supporting). **Miriam L. Conway:** Formal analysis (lead); writing – original draft (equal); writing – review and editing (equal). **Irene Ctori:** Formal analysis (equal); writing – original draft (equal); writing – review and editing (equal). **Rakhee Shah:** Formal analysis (equal); writing – original draft (equal); writing – review and editing (equal). **Catherine M. Suttle:** Conceptualization (equal); formal analysis (equal); methodology (equal); project administration (equal); supervision (lead); writing – original draft (lead); writing – review and editing (equal).

## FUNDING INFORMATION

No funding was received in support of this research.

## CONFLICT OF INTEREST

None of the authors have conflicts of interest to declare.

## Supporting information


Appendix S1
