# Supplementary material for: Refractive prescribing for preschool children by optometrists in England
Source: Ophthalmic Physiol Opt. 2022 Sep 13;43(1):6–16. doi: 10.1111/opo.13050 (PMC10087314; doi:10.1111/opo.13050)
Supplement: Supplementary file 1 — Appendix S1 [file OPO-43-6-s001.pdf]

Section A

About your work in community practice

Over the past year, in what type of optometric community practice(s) have you worked? For each practice type, please indicate the approximate percentage of your time spent in optometric community practice spent over the past year **(should equal 100% in total)**

	0	10	20	30	40	50	60	70	80	90	100
Multiple											
Independent											
Locum											
Hospital											

Approximately how many years have you worked in community practice excluding significant periods of leave?

	0	10	20	30	40	50	60	70	80	90	100
Years in Practice											

Do you have a postgraduate qualification or previous experience specific to Paediatric optometry?

Yes

No

If you answered 'Yes', please provide details of the type of qualification, registration or experience (e.g. Postgraduate Certificate, MSc or BABO, previous hospital paediatric clinic) and year completed:

In an average month, on how many children of ages 0-2 years and 3-4 years do you carry out an eye examination?

	Ages of child tested	
	0-2 years	3-4 years
None	☐	☐
1 to 5	☐	☐
6 to 20	☐	☐
Over 20	☐	☐

On what % of children aged 0 to 2 years and 3 to 4 years that you examine do you routinely perform a cycloplegic refraction? (If you answered 'None' above, please leave at 0%)

Please use the sliding scale:

	0	10	20	30	40	50	60	70	80	90	100
What % of 0-2 year olds?											
What % of 3-4 year olds?											

Section B

About your prescribing habits

The next questions each present a case study on which your answer should be based.
The option 'Prescribe full' means prescribe the full refractive correction;
'Prescribe reduced' means prescribe a correction less than the full refractive error;
'Refer hospital' means refer the patient to a hospital eye department;
'Refer optometrist' means refer to another optometrist specialising in paediatrics/COSI/community enhanced service scheme;
and 'No action' means no prescription or referral at this stage.

This question is based on the following case study:

An asymptomatic, 1 -year-old normally developing child presents for their first eye examination. There is no relevant family history. Internal and external eyes are healthy. Cover test shows orthophoria.

Each row in the table shows a hypothetical refractive error for this patient, three hyperopic and two myopic. Please assume that unaided acuity is equal RE and LE and is within normal limits for age. Please consider each row, and indicate the management option you would be most likely to use.

	Prescribe full	Prescribe reduced	Refer hospital	Refer optometrist	No action
R+2.00DS L+2.00DS	☐	☐	☐	☐	☐
R+5.00DS L+5.00DS	☐	☐	☐	☐	☐
R+8.00DS L+8.00DS	☐	☐	☐	☐	☐
R-2.00DS L-2.00DS	☐	☐	☐	☐	☐
R-5.00DS L-5.00DS	☐	☐	☐	☐	☐

If you would prescribe a reduced prescription, please state the reduced prescription here and briefly explain why (this box expands as you write):

This question is based on the following case study:

An asymptomatic, 1-year-old normally developing child presents for their first eye examination. There is no relevant family history. Internal and external eyes are healthy. Cover test shows orthophoria. Please assume that unaided acuity is within normal limits RE and at least two lines poorer LE.

Each row in the table presents a hypothetical refractive error for this patient. Please consider the case and indicate the management option you would be most likely to use.

	Prescribe full	Prescribe reduced	Refer hospital	Refer optometrist	No action
R+1.00DS L+3.00DS	☐	☐	☐	☐	☐
R+1.00DS L+5.00DS	☐	☐	☐	☐	☐

If you would prescribe a reduced prescription, please state the reduced prescription here and briefly explain why (this box expands as you write):

This question is based on the following case study:

An asymptomatic, 1-year-old normally developing child presents for their first eye examination. There is no relevant family history. Internal and external eyes are healthy. Cover test shows orthophoria.

Each row in the table presents a hypothetical refractive error for this patient. Assume that for both of these refractive errors unaided acuity is approximately equal RE and LE, for the first of these (-1.00DC) it is within normal limits and for the second (-3.00DC) it is at least two lines poorer.Please consider the case and indicate the management option you would be most likely to use.

	Prescribe full	Prescribe reduced	Refer hospital	Refer optometrist	No action
RE+1.00/-1.00x180<br>LE+1.00/-1.00x180	☐	☐	☐	☐	☐
RE+1.00/-3.00x180<br>LE+1.00/-3.00 x 180	☐	☐	☐	☐	☐

If you would prescribe a reduced prescription, please state the reduced prescription here and briefly explain why (this box expands as you write):

This question is based on the following case study:

An asymptomatic, 3-year-old normally developing child presents for their first eye examination. There is no relevant family history. Internal and external eyes are healthy. Cover test shows orthophoria.

Each row in the table shows a hypothetical refractive error for this patient, three hyperopic and two myopic. Please assume that unaided acuity is equal RE and LE and is within normal limits for age. Please consider the case and indicate the management option you would be most likely to use.

	Prescribe full	Prescribe reduced	Refer hospital	Refer optometrist	No action
R +2.00DS L +2.00DS	☐	☐	☐	☐	☐
R +5.00DS L +5.00DS	☐	☐	☐	☐	☐
R +8.00DS L +8.00DS	☐	☐	☐	☐	☐
R -2.00DS L -2.00DS	☐	☐	☐	☐	☐
R -5.00DS L -5.00DS	☐	☐	☐	☐	☐

If you would prescribe a reduced prescription, please state the reduced prescription here and briefly explain why (this box expands as you write):

This question is based on the following case study:

An asymptomatic, 3-year-old normally developing child presents for their first eye examination. There is no relevant family history. Internal and external eyes are healthy. Cover test shows orthophoria. Assume that unaided acuity is within normal limits RE and at least two lines poorer LE.

Each row in the table presents a hypothetical refractive error for this patient. Please consider the case and indicate the management option you would be most likely to use.

	Prescribe full	Prescribe reduced	Refer hospital	Refer optometrist	No action
R +1.00DS L +3.00DS	☐	☐	☐	☐	☐
R +1.00DS L +5.00DS	☐	☐	☐	☐	☐

If you would prescribe a reduced prescription, please state the reduced prescription here and briefly explain why (this box expands as you write):

This question is based on the following case study:

An asymptomatic, 3-year-old normally developing child presents for their first eye examination. There is no relevant family history. Internal and external eyes are healthy. Cover test shows orthophoria.

Each row in the table presents a hypothetical refractive error for this patient. Assume that for both of these refractive errors unaided acuity is approximately equal RE and LE; for the first of these (-1.00DC) it is within normal limits and for the second (-3.00DC) it is at least two lines poorer. Please consider the case and indicate the management option you would be most likely to use.

	Prescribe full	Prescribe reduced	Refer hospital	Refer optometrist	No action
RE +1.00/-1.00 x 180<br>LE +1.00/-1.00 x 180	☐	☐	☐	☐	☐
RE +1.00/-3.00 x 180<br>LE +1.00/-3.00 x 180	☐	☐	☐	☐	☐

If you would prescribe a reduced prescription, please state the reduced prescription here and briefly explain why (this box expands as you write):

Section C

About the basis for your spectacle prescribing in pre-school children

Apart from signs, symptoms and history, other sources of information or guidance may be used to make decisions on spectacle prescribing.

Please use the scale below to indicate the extent to which you use each of these sources of information or guidance as a basis for your decisions on spectacle prescribing for pre-school children.

Please select one option for each source, from 'Never' to 'All of the time'.

	Never	Rarely	Sometimes	Often	All of the Time
Colleagues	☐	☐	☐	☐	☐
Postgraduate or continuing education	☐	☐	☐	☐	☐
College of Optometrists guidance	☐	☐	☐	☐	☐
Undergraduate education	☐	☐	☐	☐	☐
Peer-reviewed research	☐	☐	☐	☐	☐
Experience	☐	☐	☐	☐	☐
Internet search (e.g. Google)	☐	☐	☐	☐	☐
Cochrane library	☐	☐	☐	☐	☐

Others (please state which other sources you use as a basis for these decisions):
